# Iron depletion is a novel therapeutic strategy to target cancer stem cells

**DOI:** 10.18632/oncotarget.21846

**Published:** 2017-10-12

**Authors:** Takayuki Ninomiya, Toshiaki Ohara, Kazuhiro Noma, Yuki Katsura, Ryoichi Katsube, Hajime Kashima, Takuya Kato, Yasuko Tomono, Hiroshi Tazawa, Shunsuke Kagawa, Yasuhiro Shirakawa, Fumiaki Kimura, Ling Chen, Tomonari Kasai, Masaharu Seno, Akihiro Matsukawa, Toshiyoshi Fujiwara

**Affiliations:** ^1^ Department of Gastroenterological Surgery, Okayama University Graduate School of Medicine, Dentistry and Pharmaceutical Sciences, Okayama, Japan; ^2^ Department of Pathology and Experimental Medicine, Okayama University Graduate School of Medicine, Dentistry and Pharmaceutical Sciences, Okayama, Japan; ^3^ Shigei Medical Research Institute, Okayama, Japan; ^4^ Center for Innovative Clinical Medicine, Okayama University Hospital, Okayama, Japan; ^5^ Department of Internal Medicine, Tamano General Hospital, Okayama, Japan; ^6^ Department of Medical and Bioengineering Science, Okayama University Graduate School of Natural Science and Technology, Okayama, Japan; ^7^ Department of Pathology, Tiajin Central Hospital of Gynecology Obstetrics, Tianjin, People's Republic of China

**Keywords:** cancer stem cells, induced pluripotent stem cells, iron chelators, stemness

## Abstract

Adequate iron levels are essential for human health. However, iron overload can act as catalyst for the formation of free radicals, which may cause cancer. Cancer stem cells (CSCs), which maintain the hallmark stem cell characteristics of self-renewal and differentiation capacity, have been proposed as a driving force of tumorigenesis and metastases. In the present study, we investigated the role of iron in the proliferation and stemness of CSCs, using the miPS-LLCcm cell model. Although the anti-cancer agents fluorouracil and cisplatin suppressed the proliferation of miPS-LLCcm cells, these drugs did not alter the expression of stemness markers, including Nanog, SOX2, c-Myc, Oct3/4 and Klf4. In contrast, iron depletion by the iron chelators deferasirox and deferoxamine suppressed the proliferation of miPS-LLCcm cells and the expression of stemness markers. In an allograft model, deferasirox inhibited the growth of miPS-LLCcm implants, which was associated with decreased expression of Nanog and Sox2. Altogether, iron appears to be crucial for the proliferation and maintenance of stemness of CSCs, and iron depletion may be a novel therapeutic strategy to target CSCs.

## INTRODUCTION

According to the cancer stem cell (CSC) hypothesis, cancer tissues contain a low percentage of CSCs, which have the ability to differentiate and self-renew [1, 2]. CSCs are resistant to chemotherapy and radiation therapy, and are strongly implicated in relapse after treatment [3–5]. Although CSCs are considered to be important targets therapeutically, clinically effective CSC targeting strategies have not yet been established, at least partly because the low number and heterogeneity of CSCs in cancer tissues makes the evaluation of therapy difficult. Recently, a new CSC model was established from mouse induced pluripotent stem cells (miPS cells) at Okayama University [6]. This CSC model, referred to as miPS-LLccm, was epigenetically induced from miPS cells by culturing in Lewis lung carcinoma conditioned medium for 4 weeks. miPS-LLCcm cells maintain expression of stemness markers, self-renew, and are pluripotent. miPS-LLCcm cells express Nanog-driven GFP in the undifferentiated state, and also express the Yamanaka factors, Sox2, c-Myc, Oct3/4 and Klf4, which are characteristic of pluripotent stem cells, and are also CSC markers associated with tumorigenesis and resistance to therapy [7–10]. *In vivo*, miPS-LLCcm cells form tumors with a malignant phenotype including cytokeratin positive glands, high nuclear to cytoplasmic ratio, severe nuclear atypia, multiple pathological mitotic figures and hyper-vascularization. Importantly, miPS-LLCcm cells have been used as an organ specific CSC model and for analysis of the CSC microenvironment [11, 12]. Although miPS-LLCcm require induction in the context of organ specific CSC models, the system is a tractable and convenient approach for the evaluation of CSC marker expression and the efficacy of new CSC targeted therapies.

Iron plays a key role in critical cellular processes, such as energy production and DNA synthesis. Although adequate iron levels are essential for human health, iron overload is known to cause some types of cancer [13, 14]. We previously demonstrated that iron depletion had an anti-cancer effect on some types of malignancies [15–17], potentially reflecting an important role for iron in regulating the cancerous niche including stemness, and in turn suggesting that iron depletion may be an effective therapeutic option for cancer treatment. In this study, we studied the efficacy of iron depletion therapy targeting CSCs using the iron chelators, deferasirox and deferoxamine.

## RESULTS

### Iron supply enhances the proliferation of CSCs and the expression of stemness markers is maintained

**Figure 1:**
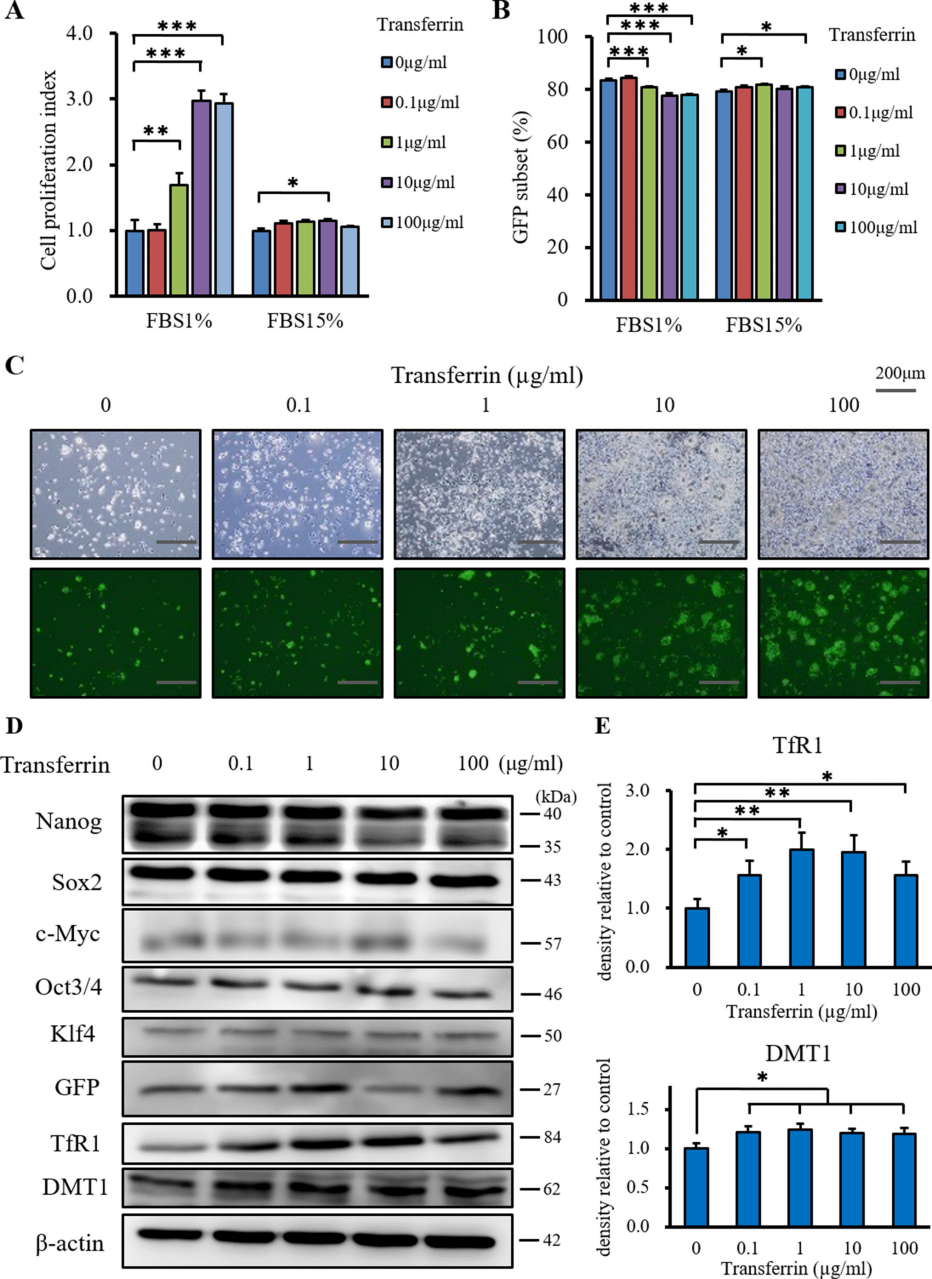
The additional effect of transferrin (Holo) on miPS-LLCcm cells *in vitro* (**A**) Iron depletion and iron rich conditions were simulated by iron free medium and 1% or 15% FCS, respectively. Cell proliferation was measured using the XTT assay after incubation with transferrin for 48 hours at 37°C. Transferrin promoted the proliferation of miPS-LLCcm cells in iron depleted conditions (FCS1%), but not in iron rich conditions (FCS15%). Data are represented as average ± S.E.M. (*n* = 5). ^**^: *p* < 0.01, ^***^: *p* < 0.001. (**B**) GFP subsets of miPS-LLCcm cells were measured by flow cytometry after incubation with transferrin for 48 hours at 37°C. Transferrin did not affect the GFP subsets of miPS-LLCcm cells. Data are represented as average ± S.E.M. (*n* = 5). (**C**) Normal and fluorescence microscopy findings showed that transferrin promoted the proliferation of GFP positive and negative cells. (**D**) Cultured miPS-LLCcm cells were treated with different concentrations of transferrin for 48 hours in iron depleted conditions. Cells were then harvested and total protein was analyzed for expression of the indicated proteins. Transferrin did not affect the expression of stemness markers and promoted the expression of TfR1 and DMT1. (**E**) Densitometry analysis of TfR1 and DMT1. The level of TfR1 and DMT1 expressed by non-treated cells was set at 100%. Data are represented as average ± S.E.M. (*n* = 3). ^*^p < 0.05, ^**^p < 0.01.

We examined the *in vitro* effect of iron supply on CSC proliferation using the XTT cell proliferation assay. We simulated starvation conditions of low growth factor and iron concentrations using iron free DMEM plus 1% FCS. The normal conditions used were iron free DMEM plus 15% FCS. Under starvation conditions, transferrin (Holo) almost tripled CSC proliferation compared with control conditions (Figure 1A). Transferrin (Holo) did not enhance the proliferation of CSCs under normal conditions. Although there was a similar trend for differentiated murine cancer cells (Supplementary Figure 1B), murine normal fibroblasts were not affected (Supplementary Figure 1C). Flow cytometry and fluorescence microscopy showed that the GFP subset was not affected by iron supply (Figure 1B and 1C). The stemness markers, Nanog, Sox2, c-Myc, Oct3/4 and Klf4, were not affected by iron supply in western blot analysis (Figure 1D), suggesting that iron supply did not affect the stemness marker phenotype of the CSCs. The expression of Nanog was also unaffected by iron supply in miPS cells (Supplementary Figure 1D) and sphere formation and GFP fluorescence were unchanged (Supplementary Figure 1E). The expression of the transferrin receptor 1 (TfR1) and DMT1 were increased by iron supply in CSCs (Figure 1D and 1E). This result indicated that the CSCs responded to the iron supply by increasing iron uptake under starvation conditions. Together, iron supply enhanced the proliferation of CSCs, while the expression of stemness markers was maintained.

### Iron chelators suppress the proliferation of CSCs

**Figure 2:**
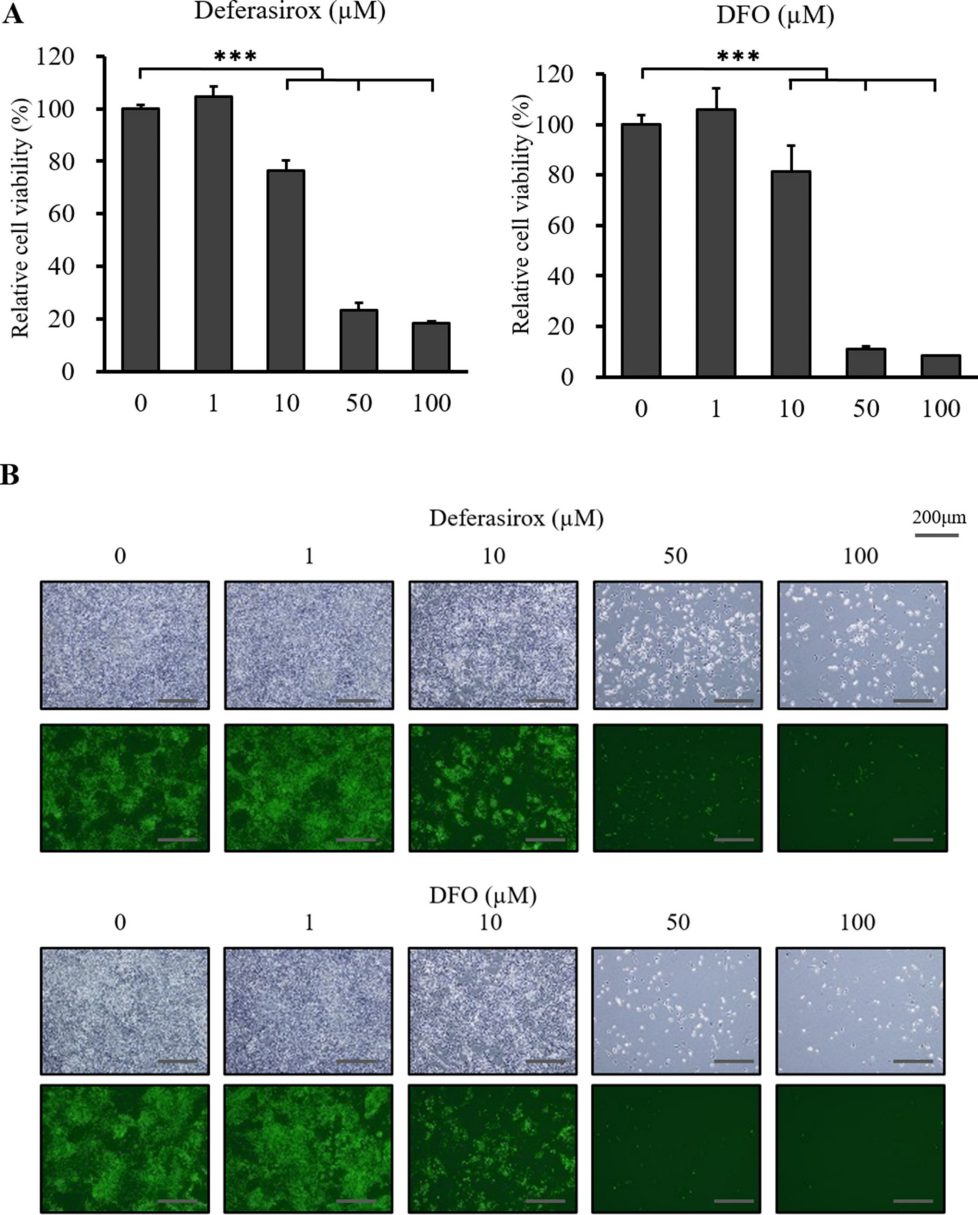
The inhibitory effect of deferasirox and DFO on miPS-LLCcm cells *in vitro* (**A**) Cultured miPS-LLCcm cells were treated with different concentrations of deferasirox and DFO for 48 hours and cell viability was then evaluated using the XTT assay. Cell viability in the absence of treatment was set at 100%. Results are means of 3 independent experiments. Data are represented as average ± S.E.M. (*n* = 5). ^***^p < 0.001. (**B**) Normal and fluorescence microscopy findings showed that numbers of GFP positive and negative cells were decreased in a dose dependent manner.

We examined the *in vitro* antiproliferative activity of the iron chelators, deferasirox and DFO, against the miPS-LLCcm cells using XTT cell viability assay. CSCs were incubated with deferasirox and DFO in normal medium with 15% FCS for 48 hours. Deferasirox and DFO suppressed the proliferation of CSCs in a dose dependent manner (Figure 2A). Fluorescence microscopy also showed that deferasirox and DFO decreased the numbers of GFP positive cells in a dose dependent manner (Figure 2B). The iron chelators suppressed the proliferation of the differentiated mouse cancer cells, colon26 and 4T1, at 48 hours (Supplementary Figure 2A). However, the chelators did not show strong antiproliferative activity against the normal fibroblast cell lines, MEF and NIH-3T3, at 48 hours (Supplementary Figure 2B). IC50 analyses showed that deferasirox had a stronger antiproliferative effect than DFO (Table 1). These results indicated that the chelators had strong antiproliferative effects against CSCs and differentiated cancer cells, while normal fibroblasts were not affected.

**Table 1:** IC50 of deferasirox and DFO for miPS-LLCcm cells

Chelator	IC50 (μM)
Deferasirox	42.0
DFO	47.0

IC50 values (μM) for deferasirox and DFO after a 48-hour, 37°C incubation using the miPS-LLCcm CSC model. Data represent the mean ± S.E.M. (*n* = 3).

### Iron chelators suppress the proliferation of CSCs by induction of apoptosis and cell cycle arrest

**Figure 3:**
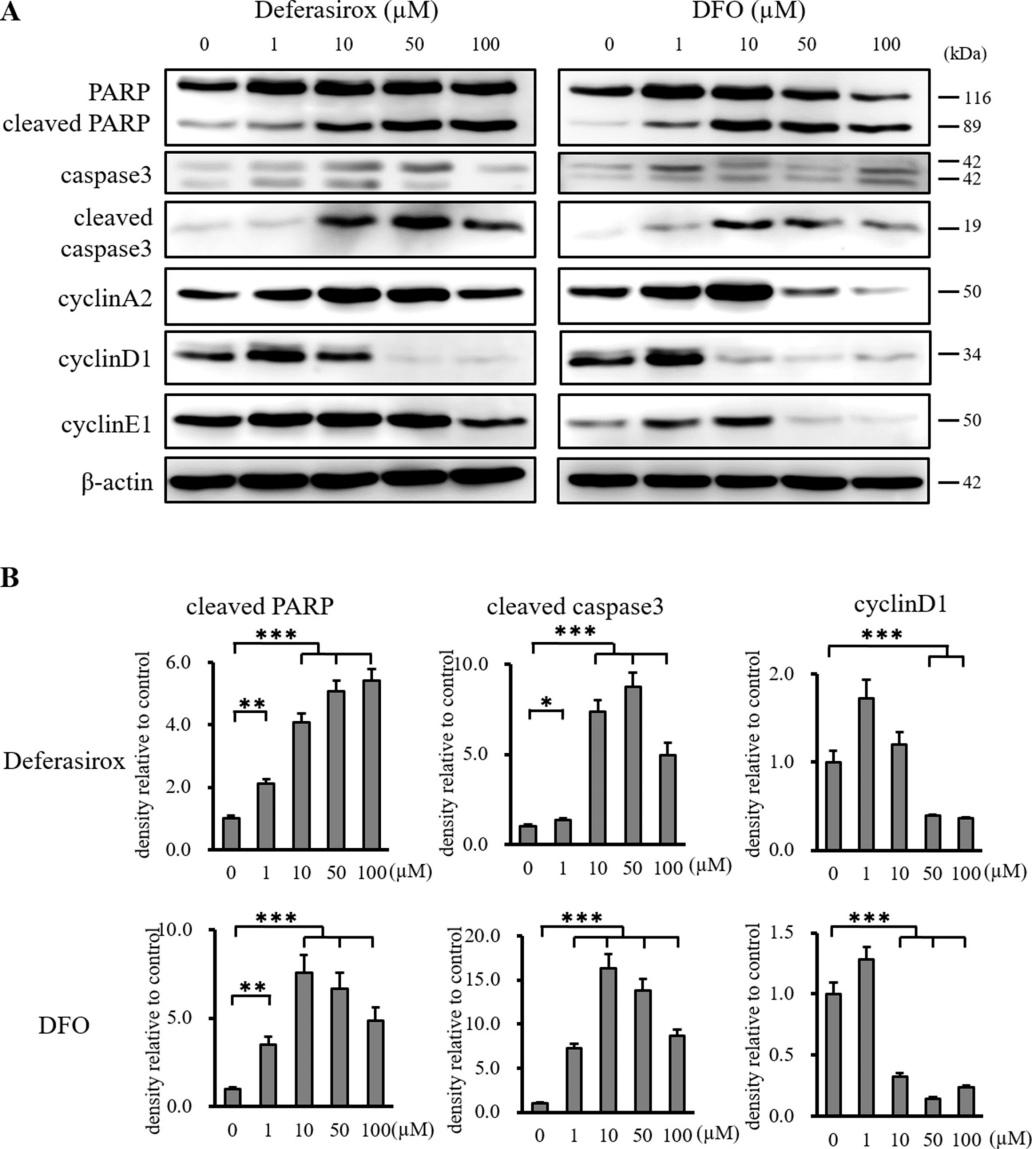
Western blot analysis of apoptosis and cell cycle related proteins in miPS-LLCcm cells (**A**) Cultured miPS-LLCcm cells were treated with different concentrations of deferasirox and DFO for 48 hours. Cells were then harvested and total protein was analyzed for expression of the indicated proteins. Deferasirox and DFO induced the apoptosis related markers, cleaved caspase 3 and cleaved PARP, and decreased the cell cycle related marker, cyclin D1. (**B**) Densitometry analysis of the western blots also showed deferasirox and DFO significantly induced cleaved caspase 3 and cleaved PARP, and decreased cyclin D1 and cyclin E1. Data are represented as average ± S.E.M. (*n* = 3) ^*^p < 0.05, ^**^p < 0.01, ^***^p < 0.001.

We examined the mechanisms underlying the antiproliferative effects of the iron chelators on CSCs. The expression of cleaved PARP and cleaved caspase 3 was increased by iron chelators in the western blot and densitometry analyses, which indicated that iron chelators induced apoptosis (Figure 3A and 3B). The induction of apoptosis was confirmed by TUNEL staining (Supplementary Figure 3A). The expression of cyclin D1, a key cell cycle control protein, was decreased by the iron chelators, which indicated that iron chelators induced cell cycle arrest in CSCs. Although the expression of cyclin A2 and cyclin E1 was also suppressed by DFO, Deferasirox did not significantly suppress their expression (Supplementary Figure 3B). These results indicated that iron chelators suppressed the proliferation of CSCs by induction of apoptosis and cell cycle arrest.

### Iron chelators suppress the expression of stemness markers, including Nanog and Yamanaka factors, in the CSC model

**Figure 4:**
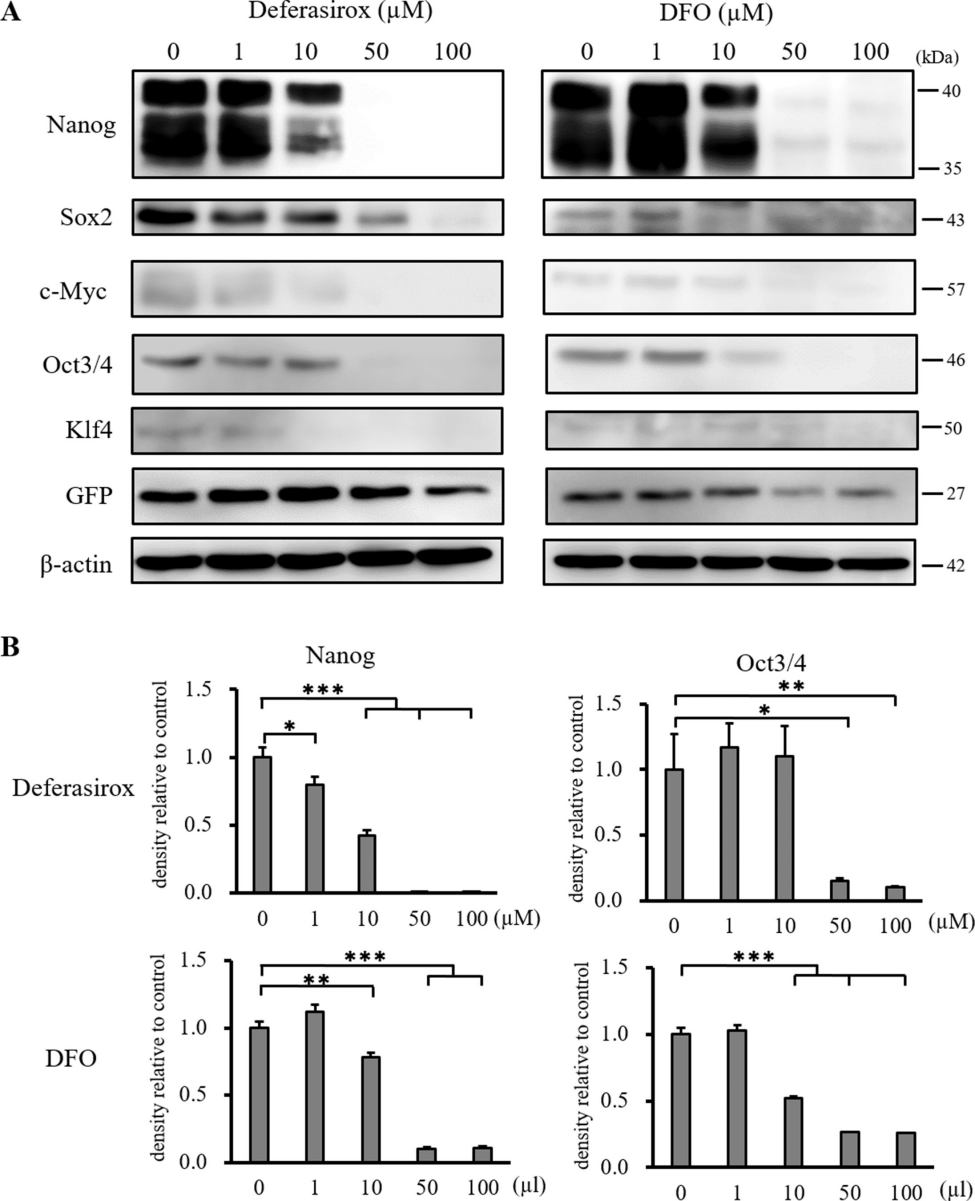
Western blot analysis of stemness marker expression in miPS-LLCcm cells (**A**) Cultured miPS-LLCcm cells were treated with different concentrations of deferasirox and DFO for 48 hours. Cells were then harvested and total protein was analyzed for the expression of indicated proteins. Deferasirox and DFO decreased the expression of stemness markers. (**B**) Densitometry analysis of the western blots also showed deferasirox and DFO significantly decreased the expression of the stemness markers, Nanog and Oct3/4. Data are represented as average ± S.E.M. (*n* = 3) ^*^p < 0.05, ^**^p < 0.01, ^***^p < 0.001.

Western blot analysis revealed that the iron chelators significantly suppressed the expression of stemness markers, Nanog and Oct3/4 (Figure 4A and 4B). Although the expression of Sox2, c-Myc, and Klf4 was also suppressed by deferasirox in a dose dependent manner, DFO did not significantly suppress these markers (Supplementary Figure 4). These results revealed that iron chelators suppressed the expression of CSC stemness markers, and the effect of deferasirox was stronger than that of DFO.

### 5-FU and cisplatin do not suppress the expression of stemness markers in CSCs

**Figure 5:**
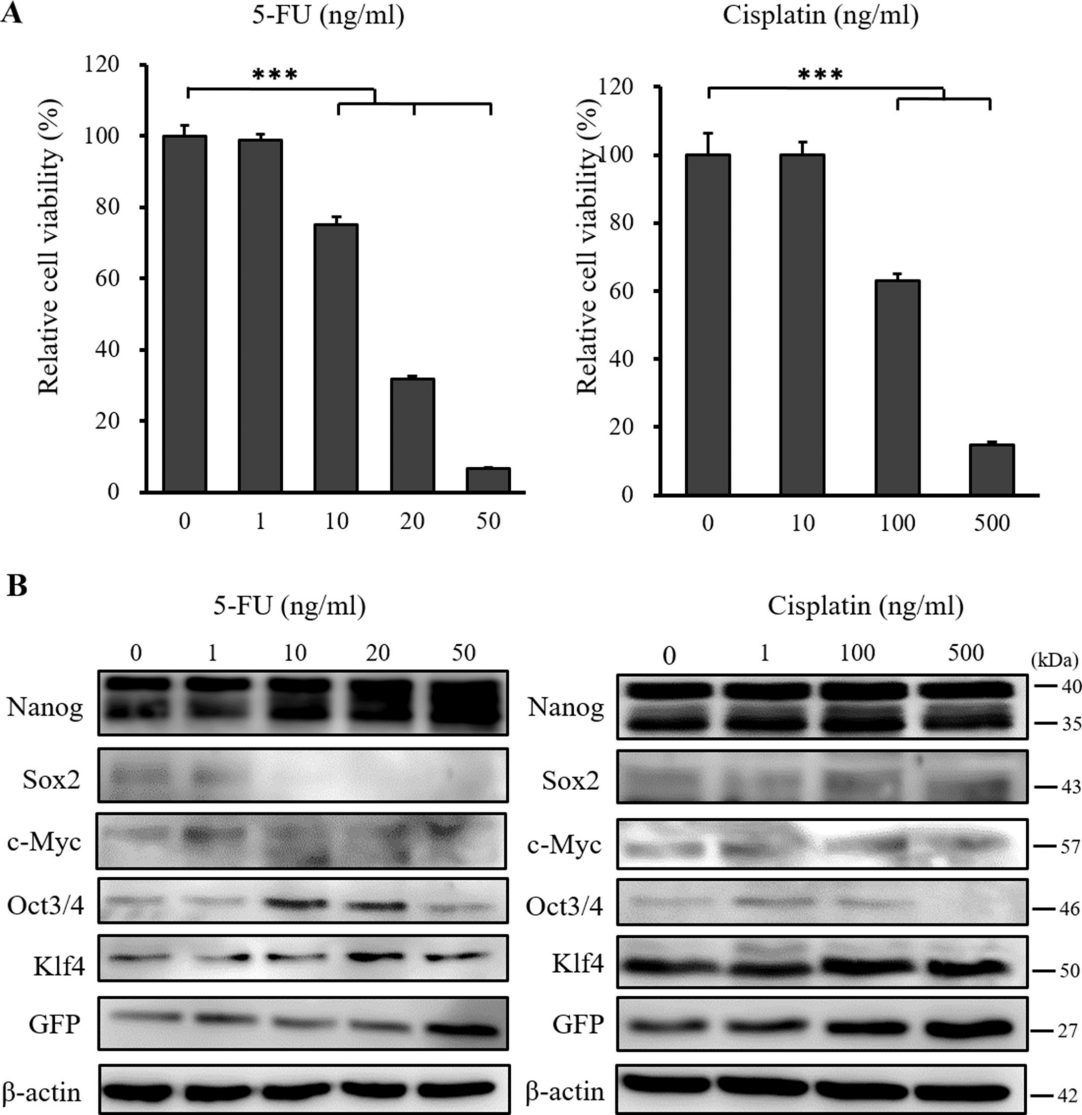
XTT cell proliferation assay and western blot analysis of miPS-LLCcm cells following 5-FU and cisplatin treatment (**A**) Cultured miPS-LLCcm cells were treated with different concentrations of 5-FU and cisplatin for 48 hours, and cell viability was then evaluated using the XTT assay. 5-FU and cisplatin suppressed the proliferation of miPS-LLCcm cells in a dose dependent manner. Cell viability in the absence of treatment was set at 100%. Results are means of 3 independent experiments. Data are represented as average ± S.E.M. (*n* = 5). ^***^p < 0.001. (**B**) Cultured miPS-LLCcm cells were treated with different concentrations of 5-FU and cisplatin for 48 hours. Cells were then harvested and total protein was analyzed for expression of the indicated proteins. Western blot analysis showed that 5-FU and cisplatin did not suppress the expression of stemness markers.

To address the effects of standard chemotherapy on the CSC model, we examined cell viability and the expression of stemness markers following treatment with 5-FU and cisplatin. 5-FU and cisplatin suppressed the viability of CSCs in a dose dependent manner (Figure 5A), but did not suppress the expression of stemness markers (Figure 5B). GFP positive cells were also detected by fluorescence microscopy after treatment (data not shown) and the expression of GFP did not decrease (Supplementary Figure 4B). These results indicated that 5-FU and cisplatin acted only on differentiated cells and not on cells expressing stemness markers, suggesting that the suppression of stemness markers is a unique function of iron chelators.

### Iron chelators suppress tumor growth and expression of stemness markers

**Figure 6:**
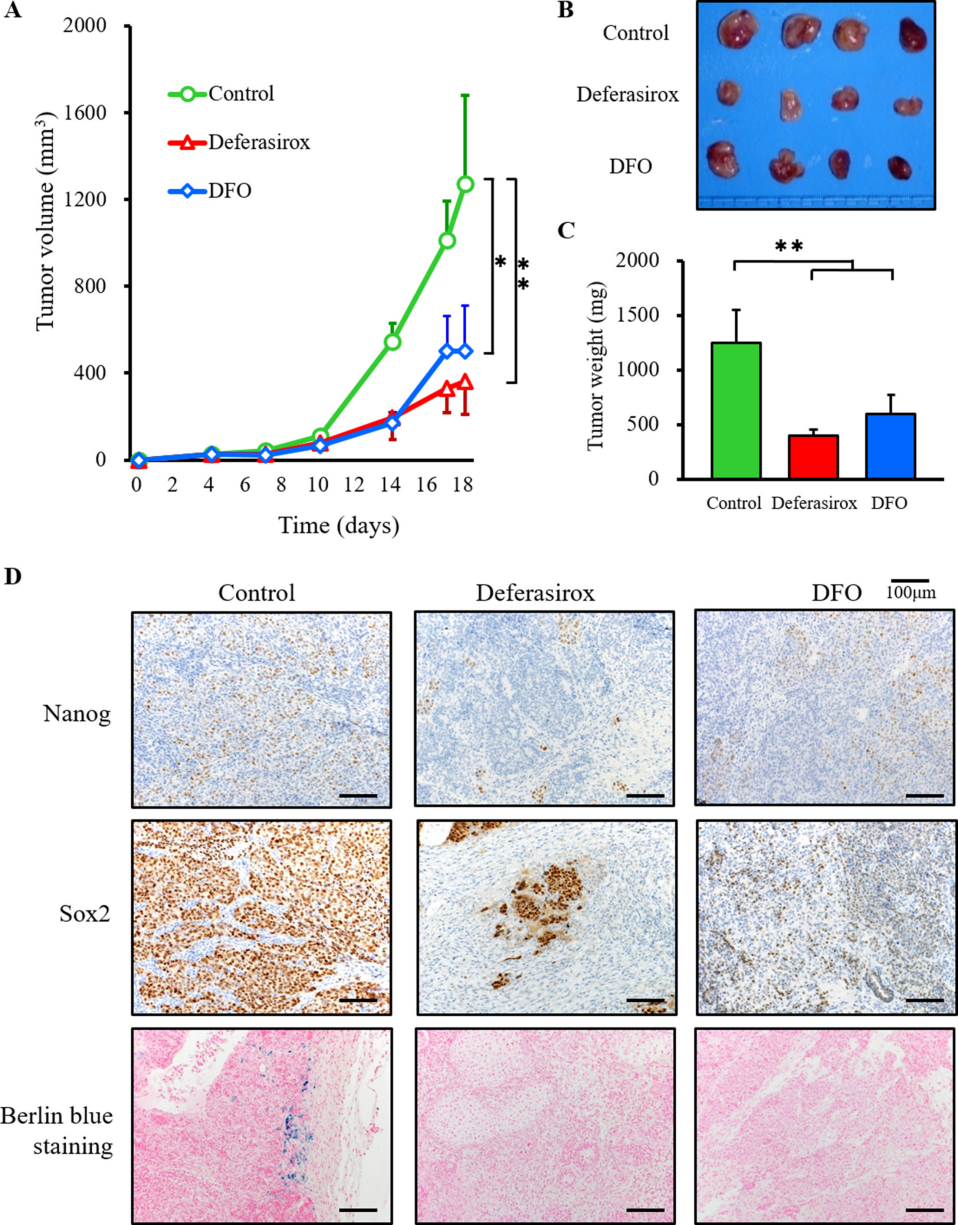
Iron chelators suppressed the growth of CSC tumor allografts and suppressed stemness marker expression (**A**) miPS-LLCcm cells (1 × 10^6^ per animal) were implanted subcutaneously into the right back flank of mice and treatment commenced 5 days after tumor injection. Deferasirox and DFO (30 mg/kg locally, given by injection 5 days per week for 14 days) effectively inhibited the growth of miPS-LLCcm allografts *in vivo*. ^*^p < 0.05, ^**^p < 0.01. (**B**) Macroscopic images showed that tumors in both deferasirox and DFO groups were smaller than in the control group. (**C**) Tumor weight was significantly decreased in the deferasirox and DFO groups. Data are represented as average ± S.E.M. (*n* = 4). ^**^p < 0.01. (**D**) Resected tumors were analyzed for Nanog and SOX2 expression by immunohistochemistry, and Fe^3+^ by Berlin blue staining. Nanog and SOX2 were suppressed in the deferasirox and DFO groups. Berlin blue staining was not detected in the deferasirox and DFO groups.

To address the suppression of proliferation and stemness markers *in vivo*, we employed a miPS-LLCcm CSC tumor allograft model using BALB/c nude mice. The chelators were administered directly according to clinical dose guidelines at 30 mg/kg for 5 days in a week. After 18 days of local treatment with the vehicle control (PBS) the tumor allografts reached an average volume of 1270.82 ± 411.17 mm^3^. However, the iron chelators significantly decreased tumor growth (deferasirox 360.27 ± 148.84 mm^3^; DFO 502.53 ± 207.45 mm^3^, Figure 6A and 6B). Tumor weight was also significantly decreased in the iron chelator groups (tumor weight: control vs deferasirox vs DFO: 1250 ± 300 mg vs 600 ± 180 mg vs 400 ± 50 mg) (Figure 6C). The body weights of treated mice were not significantly different to controls, which indicated that iron chelators did not induce serious side effects (Supplementary Figure 5A). To examine the suppression of stemness markers, tumors were collected for immunohistochemistry. Deferasirox and DFO suppressed the expression of GFP and stemness markers, including Nanog, Sox2, c-Myc, Oct3/4 (Figure 6D and Supplementary Figure 5B). The blue spots of berlin blue iron staining were detectable only in the control group, which indicated that iron in the tumor was decreased by deferasirox and DFO. These results indicated that iron chelators suppressed CSC tumor growth and the expression of stemness markers *in vivo*.

## DISCUSSION

The expression of stemness markers is reported to be linked to increased malignant potential [10]. Although CSCs are thought to be associated with resistance to chemotherapy, the lack of a suitable study model to comprehensively address this question [18–20] has compromised the development of CSC therapies. In this regard, our CSC model has some significant advantages over other models, including the stable expression of stemness markers, such as Nanog and the Yamanaka factors, and the regulation of stemness marker expression by puromycin selection. This latter feature in particular, facilitates more consistent experimental procedures for evaluating the efficacy of CSC targeted therapies.

Iron overload is known to induce some types of cancer [13, 14] and iron chelators were reported to have anti-cancer effects [21, 22]. We also reported that iron depletion had therapeutic potential for cancer treatment [15, 16] and not only suppressed the proliferation of cancer cells, but also their migration and invasion abilities [17]. These findings indicated that cancer cells use iron for many functions including proliferation and metastasis, and iron may also be critical for the maintenance of CSCs. Indeed, our studies showed that iron chelators have significant antiproliferative effects on both CSCs and differentiated cancer cells, but not normal fibroblasts, possibly because CSCs and differentiated cancer cells have a higher proliferative potential than normal fibroblasts.

The expression of stemness markers on CSCs was suppressed by iron chelators, which indicated that iron is linked to the maintenance of stemness. However, the relationship between stemness and iron metabolism is still unclear. Although both iron chelators increased the expression of TfR-1, the expression of DMT-1 remained unchanged (Supplementary Figure 3C and 3D). Increased expression of TfR-1 in response to iron depletion was anticipated. However, the reasons why DMT-1 expression did not increase in parallel remain unclear. The expression of p53 tended to be increased by iron chelators (Supplementary Figure 3C), although not at higher concentrations of deferasirox (> 10 μM). Although the increase in p53 expression may reflect cell cycle arrest, further analysis is required to comprehensively address this possibility.

There are some reports that the inhibition of Nanog suppressed cancer cell proliferation and tumor growth [23, 24], and our results indicated that the role of Nanog might be similar in CSCs. 5-FU and cisplatin could not suppress the expression of stemness markers, which indicated that these standard anti-cancer drugs may not target CSCs. Thus, iron chelators may be an effective adjunct to standard chemotherapy. Moreover, iron chelators may reduce the development of resistance by suppression of the expression of stemness markers in the tumor. Further analysis of this phenomenon using orthotropic tumor models may help delineate the mechanisms underlying the development of resistance [25–27].

Deferasirox suppressed the expression of stemness markers more effectively than DFO. This is consistent with the suggestion that the chelation ability of deferasirox is stronger than DFO [28]. Although the therapeutic potential of PI3K/mTOR dual inhibitors for targeting CSCs has been reported [29, 30], this is the first description of an agent that can effectively suppress stemness marker expression, which may be a unique function of iron chelators.

Although iron chelators suppressed the expression of stemness markers, the mechanisms are currently unknown. This study may represent an important first step in the development of novel iron chelation based strategies for CSC therapy.

## MATERIALS AND METHODS

### Cell culture

Mouse induced pluripotent stem cells (miPS) were purchased from Riken Cell Bank (RIKEN BRC, Ibaraki, Japan). The Mouse Lewis lung carcinoma (LLC) cell line, mouse rectal cancer cell line (colon26) and mammary cancer cell line (4T1) were purchased from the American Type Culture Collection (ATCC, Manassas, VA, USA). We also used mouse fibroblast cell lines, MEF and NIH-3T3, that were obtained from the ATCC as representative of cells of a “normal” non-cancer phenotype. All cells were incubated at 37°C in a humidified atmosphere containing 5% CO_2_. miPS-LLCcm cells were epigenetically induced from miPS cells by culturing in Lewis lung carcinoma conditioned medium for 4 weeks [6, 11]. Cell culture dishes and multi-well plates for miPS-LLCcm culture were coated with 0.1% gelatin solution for 30 min at 37°C. miPS-LLCcm cells were maintained in DMEM containing 15% FCS, 0.1 mM NEAA, 2 mM L-Glutamine, 0.1 mM 2-mercaptoethanol, 50 U/ml penicillin and 50 U/ml streptomycin. Colon26, 4T1 MEF and NIH-3T3 cells were maintained in DMEM containing 10% FCS, 50 U/ml penicillin and 50 U/ml streptomycin.

### Puromycin selection

miPS-LLCcm cultures contain GFP positive and GFP negative cells. Nanog driven GFP cells carry the puromycin-resistance gene facilitating the selection of miPS-LLCcm CSCs by puromycin resistance. Puromycin (puromycin dihydrochloride, catalog no. p8833, Sigma-Aldrich, St. Louis, MO, USA) was dissolved in distilled water at a stock concentration of 1 μg/μl and added to the miPS-LLCcm medium at a final concentration of 1 μg/ml. After puromycin selection, GFP positive cells were purified to almost 90% homogeneity (Supplementary Figure 1A). This approach was used to purify CSCs (GFP positive cells) for all experiments. Experiments were performed when the cultures were approximately 80% confluent after puromycin selection.

### Holo-transferrin

Human Transferrin (Holo) (catalog no. 208–18971, Wako Pure Chemical Industries, Osaka, Japan) was dissolved in distilled water at a stock concentration of 1 μg/μl and used as an iron supplement. Transferrin (Holo) was dissolved in distilled water at indicated concentrations.

### Iron chelators

Deferasirox (EXJADE) and Deferoxamine (DFO, Desferal) were obtained from Novartis Pharma (Tokyo, Japan). For *in vitro* studies, DFO was dissolved in distilled water at a stock concentration of 50 mM. Deferasirox was dissolved in 100% DMSO (Sigma-Aldrich) at a stock concentration of 50 mM. DFO and deferasirox were dissolved in distilled water at indicated concentrations and directly injected.

### Chemotherapeutic agents

5-fluorouracil (5-FU) was purchased from Kyowa Kirin (Tokyo, Japan), and dissolved in distilled water. Cisplatin (Randa) was purchased from Nippon Kayaku (Tokyo, Japan) and dissolved in PBS.

### Cell proliferation assay

The XTT assay (Cell proliferation kit II, Roche Mannheim, Germany) was used to assess cell proliferation. We simulated iron depletion conditions using iron free DMEM plus 1% fetal calf serum (FCS), and iron rich conditions using iron free DMEM plus 15% FCS. The cells were incubated with holo-transferrin with 1% or 15% FCS and iron free DMEM for 48 hours. The cells were incubated with deferasirox and DFO for 48 hours at 37°C in iron free medium plus 1% or 15% FCS. All experiments used 96-well plates and each experiment was repeated at least 3 times. Optical densities were measured at 450 nm and 690 nm. We seeded the cells as follows; miPS-LLCcm (1.0 × 10^4^/well), 4T1 and colon26 (3.0 × 10^3^/well), MEFs and NIH-3T3 (1.0 × 10^4^/well).

### Flow cytometry

GFP subsets of miPS-LLCcm cells were analyzed by flow cytometry (FACSArray, Becton Dickinson, Franklin Lakes, NJ, USA) using standard techniques.

### Sphere formation assay

Single cells were plated into ultra-low attached 96-well plates (Corning Costar, Sigma-Aldrich) at a density of 2.5 × 10^4^ cells/ml. Cells were grown in serum-free miPS medium without leukemia inhibitory factor, and incubated for 72 hr.

### Western blotting

Protein was extracted from whole cells after 48 hours of incubation with medium and transferrin (Holo) or iron chelators. The concentrations of extracted protein were measured using standard protocols. Cells were lysed using cell lysis buffer (50 mmol/L Tris-HCl (pH7.4), 30 mmol/L NaCl, and 1% Triton X-100) containing protease inhibitors (cOmplete Mini, Roche Diagnostics GmbH, Basel, Switzerland). Equal amounts of total cellular proteins were separated by sodium dodecyl sulfate-polyacrylamide gel electrophoresis and transferred electrophoretically to polyvinylidene difluoride filter membranes (GE Healthcare UK Ltd, Buckinghamshire, UK). Protein samples (40 μg/lane) were separated on 8% or 12% Tris-acrylamide gels and transferred to PVDF membranes (GE Healthcare Life Sciences) according to the manufacturer's protocol. The following primary antibodies were used: rabbit polyclonal anti-Nanog antibody (catalog no. ab80892; Abcam, Cambridge, MA, USA), rabbit anti-mouse SOX2 antibody (catalog no. ab97959, Abcam), rat anti-mouse Oct3/4 antibody (catalog no. MAB1759; R&D systems, Minneapolis, MN, USA), rabbit anti-mouse c-Myc antibody (catalog no. ab32072; Abcam), rabbit anti-mouse Klf4 antibody (catalog no. ab72543, Abcam), rabbit GFP polyclonal antibody (catalog no. 2956; Cell Signaling, Danvers, MA, USA), rabbit anti transferrin receptor antibody (catalog no. ab84036; Abcam), mouse monoclonal anti-β-actin antibody (catalog no.A5441, Sigma-Aldrich), rabbit polyclonal anti-PARP antibody (catalog no. 9542; Cell Signaling), rabbit polyclonal anti-cleaved caspase-3 antibody (catalog no. 9664; Cell Signaling), rabbit polyclonal anti-caspase 3 antibody (catalog no. sc-7148; Santa Cruz, Dallas, TX, USA), rabbit monoclonal anti-Cyclin A2 antibody (catalog no. ab181591, Abcam), rabbit monoclonal anti-Cyclin D1 antibody (catalog no. ab134175, Abcam), rabbit monoclonal anti-Cyclin E1 antibody (catalog no. ab33911, Abcam), Rabbit polyclonal anti-p53 antibody (catalog no. ab131442, Abcam), Rabbit polyclonal anti- DMT1 antibody (catalog no. ab123085, Abcam). All primary antibodies were used at a 1:1000 dilution. All secondary antibodies (GE Life Sciences) were used at a 1:2500 dilution. The membranes were incubated with primary antibodies overnight at 4°C, followed by incubation with secondary antibodies. An ECL prime Western Blotting Detection Reagent (GE Healthcare UK Ltd.) was used to detect the peroxidase activity of secondary antibodies. All experiments were repeated at least 3 times. Membranes were probed for β-actin as a loading control, and all sample data values were normalized to the corresponding control data values. Densitometric analysis was performed using Image J software (NIH).

### TUNEL staining

The induction of apoptosis was assessed by TUNEL assay using the MK500 *in situ* Apoptosis Detection Kit (TAKARA BIO, Shiga, Japan).

### Tumor allograft model

All animal experiments were performed according to the Japanese Welfare and Management of Animals Act and conducted in accordance with institutional guidelines at Shigei Medical Research Institute, Okayama, Japan. All animal experiments were approved by the Ethics Review Committee for Animal Experimentation of Shigei Medical Research Institute, Okayama, Japan (#150526–1). Female BALB/c (nu/nu) mice were purchased from the CLEA (Tokyo, Japan) and were housed in sterile conditions. Experiments started when the mice were 7 weeks of age. miPS-LLCcm tumor cells in culture were harvested and resuspended in a 1:1 ratio of PBS and Matrigel (BD Biosciences, San Jose, CA, USA). Viable miPS-LLCcm cells (1.0 × 10^6^) were injected into the right back flanks of the mice subcutaneously. Tumor size was measured every 2 days. Deferasirox (30 mg/kg) and DFO (30 mg/kg) were suspended in saline and injected locally into the tumor 5 days per week. The treatment was started 5 days after inoculation (*n* = 4). Control groups received PBS that was administered by local injection 5 days per week. The tumor volume was calculated by the formula: Length × Width × Height × 0.52. At the end of the experiment, the animals were sacrificed and the tumors were excised, weighed and further processed for histological analysis. These experiments were repeated 3 times.

### Immunostaining analyses of excised tumors

The resected miPS-LLCcm tumors were fixed in 10% paraformaldehyde and embedded in paraffin prior to immunostaining analysis. Anti-Nanog antibodies, anti-SOX2 antibodies, anti-GFP antibodies, anti-c-Myc antibodies, and anti-Oct3/4 antibodies were purchased from Abcam as for western blot analysis. Pottasium Hexacyanoferrate (II) (catalog no. 28608–42) was purchased from Nacalai Tesque (Kyoto, Japan), and used for Berlin blue staining.

### Statistical analysis

Data were compared against the respective control in each experiment using Student's *t* test. Results were considered statistically significant when the *P* value was less than 0.05. Data analysis was performed using JMP ver.11 software (SAS, Cary NC27513).

## SUPPLEMENTARY MATERIALS FIGURES AND TABLES


