# Supplementary material for: Iron depletion is a novel therapeutic strategy to target cancer stem cells
Source: Oncotarget. 2017 Oct 12;8(58):98405–16. doi: 10.18632/oncotarget.21846 (PMC5716739; doi:10.18632/oncotarget.21846)
Supplement: Supplementary file 1 [file oncotarget-08-98405-s001.pdf]

Iron depletion is a novel therapeutic strategy to target cancer stem cells

SUPPLEMENTARY MATERIALS

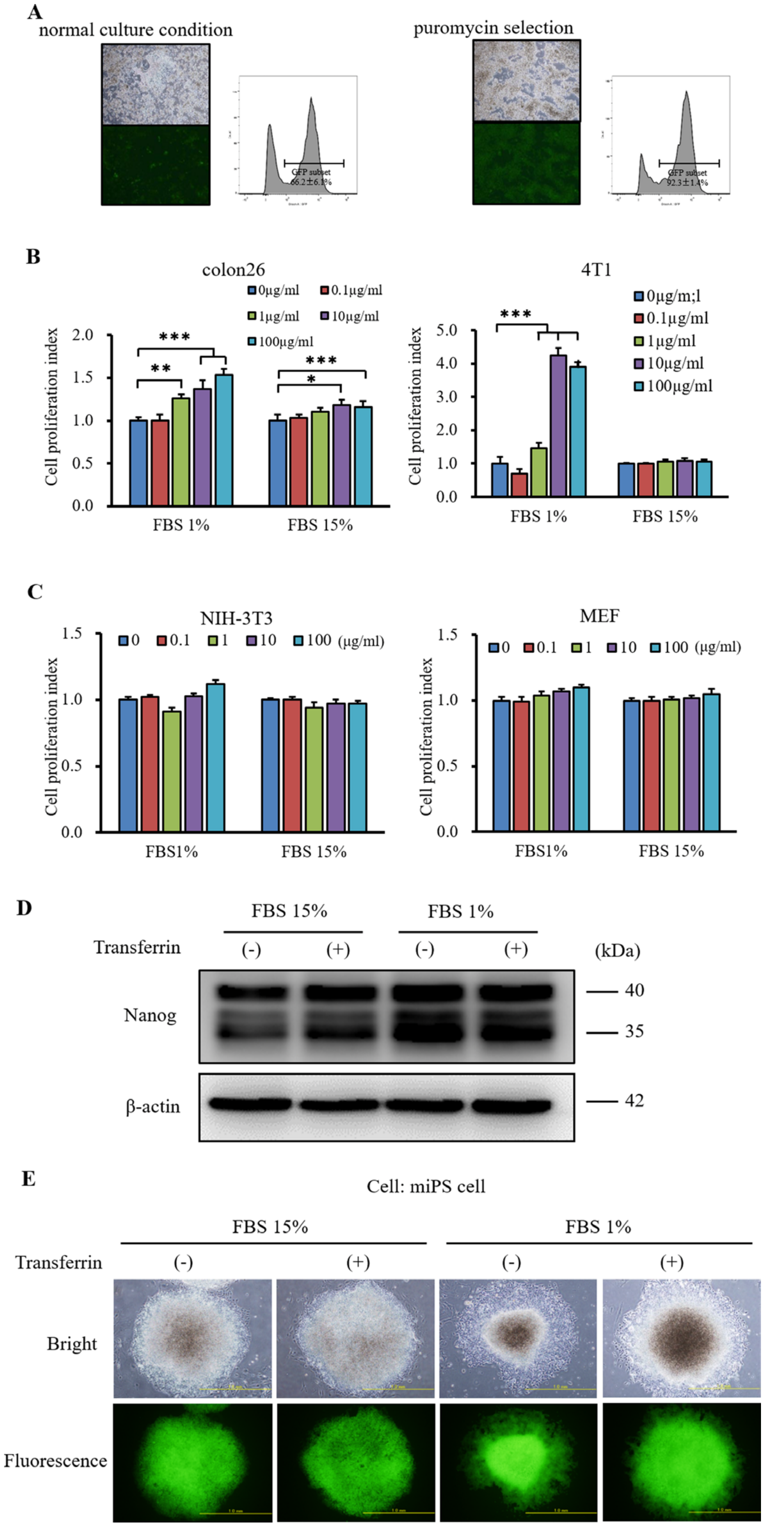

Supplementary Figure 1: (A) Puromycin selection of miPS-LLCcm CSCs. Light and fluorescence microscopy findings showed that puromycin eliminated GFP negative cells and enhanced the percentage of GFP positive cells. Flow cytometry showed that puromycin

increased the GFP subset from 66.2% ± 6.1% to 92.3% ± 1.4%. (**B, C**) Iron depletion and iron rich conditions were simulated by iron free medium and 1% or 15% FCS, respectively. Cell proliferation was measured using the XTT assay after incubation with holo-transferrin for 48 hours at 37°C. Data are represented as average ± S.E.M. ($n = 5$). $^{*}p < 0.05$, $^{**}p < 0.01$, $^{***}p < 0.001$. Transferrin promoted the proliferation of colon26 and 4T1 cells under starvation conditions (FCS 1%), but not normal conditions (FCS 15%). Data are represented as average ± S.E.M. ($n = 5$). Transferrin did not promote the proliferation of NIH-3T3 and MEF cells. (**D**) Cultured miPS cells were grown in FBS 15% and FBS 1 % with and without 50 μg/ml transferrin for 48 hours. Cells were then harvested and total protein was analyzed for expression of the indicated proteins. The expression of Nanog was maintained after transferrin administration. (**E**) Cultured miPS cells in ultra-low attachment dishes were treated with FBS 15% and FBS 1% with and without 50 μg/ml transferrin for 48 hours. Bright field and fluorescence microscopy showed that sphere formation and GFP fluorescence were maintained after transferrin administration.

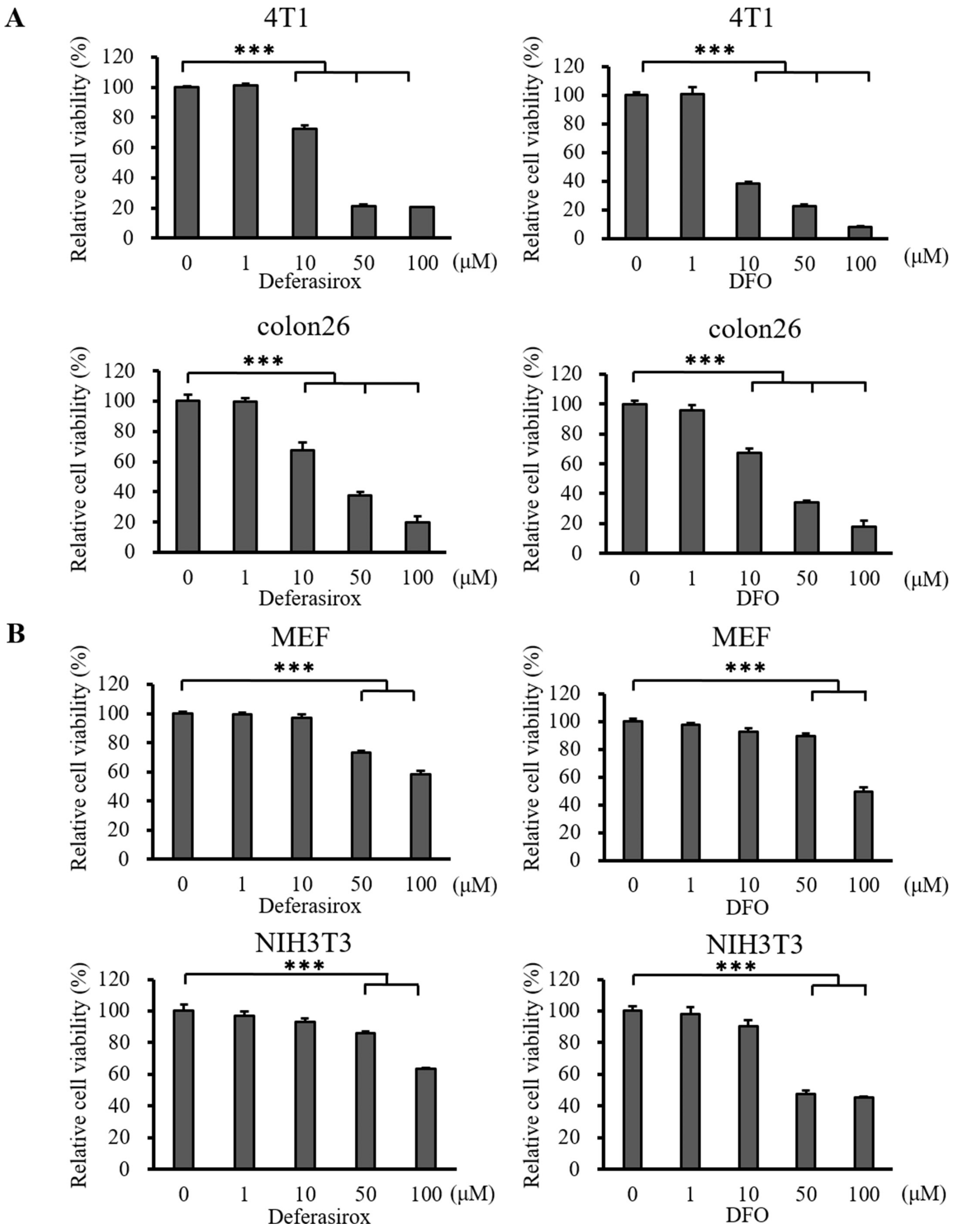

Supplementary Figure 2: The inhibitory effect of deferasirox and DFO on the growth of colon26, 4T1, NIH-3T3 and MEF cells *in vitro*. (**A**) Cultured colon26 and 4T1 cells were treated with different concentrations of deferasirox and DFO for 48 hours and cell viability was then evaluated using the XTT assay. Cell viability in the absence of treatment was set at 100%. Results are means of 3 independent experiments. Data are represented as average ± S.E.M. ($n = 5$). $^{***}p < 0.001$. Relative cell viability of colon26 and 4T1 cells was slightly decreased in a dose dependent manner. (**B**) Cultured NIH-3T3 and MEF cells were treated with different concentrations of deferasirox and DFO for 48 hours and cell viability was then evaluated using the XTT assay. Cell viability in the absence of treatment was set at 100%. Results are means of 3 independent experiments. Data are represented as average ± S.E.M. ($n = 5$). $^{***}p < 0.001$. Relative cell viability of NIH-3T3 and MEFs was slightly decreased in a dose dependent manner. The inhibitory effect of deferasirox and DFO was stronger against cancer cells than normal fibroblasts.

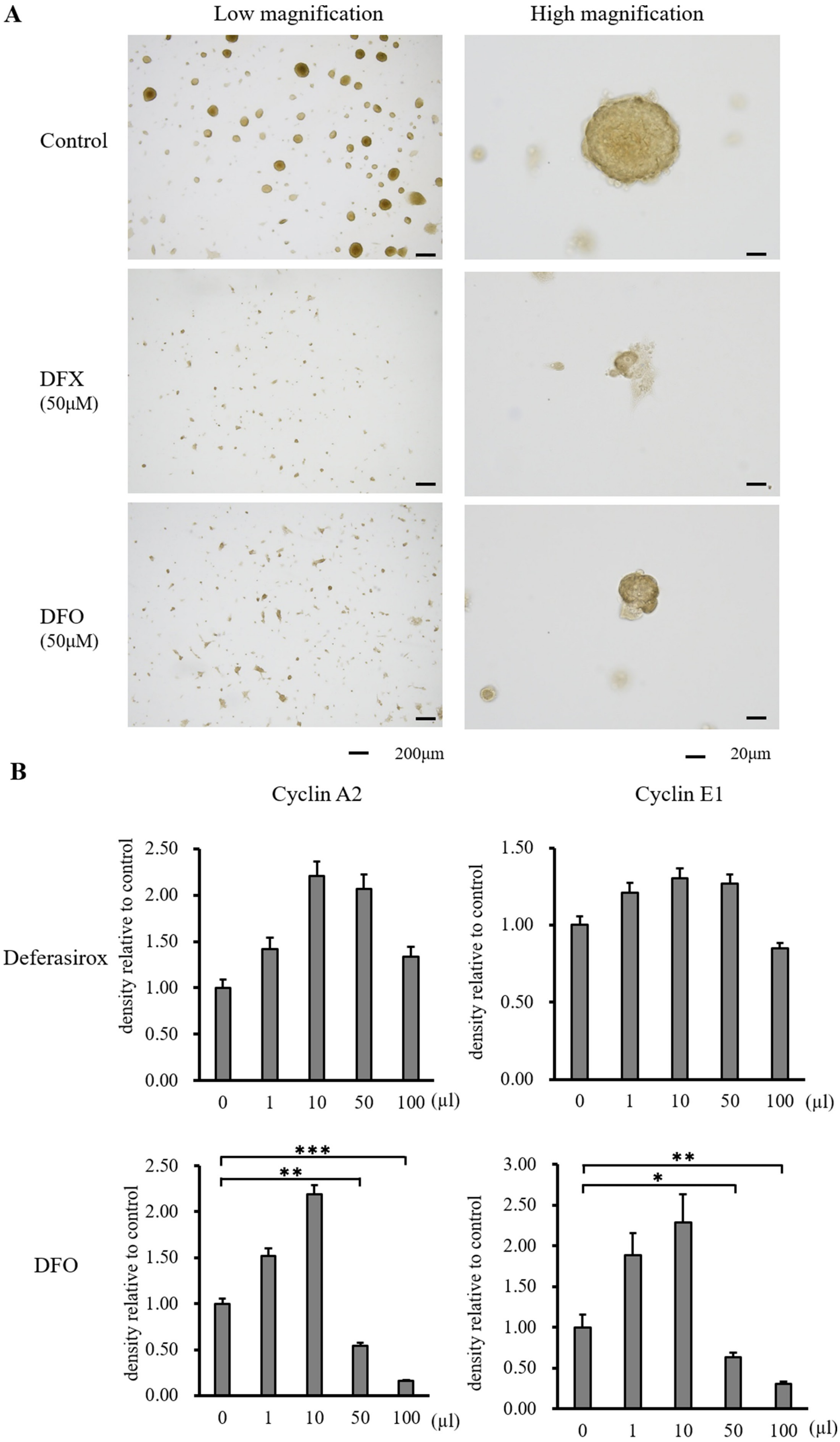

A
Low magnification
High magnification
Control
DFX
(50μM)
DFO
(50μM)
200μm
20μm
B
Cyclin A2
Cyclin E1
Deferasirox
DFO
density relative to control
0.00
0.50
1.00
1.50
2.00
2.50
3.00
0
1
10
50
100
(μl)
*
**
***

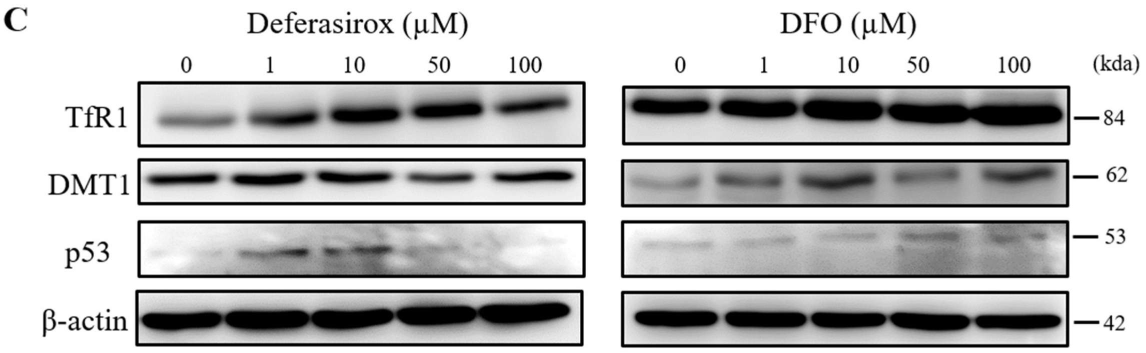

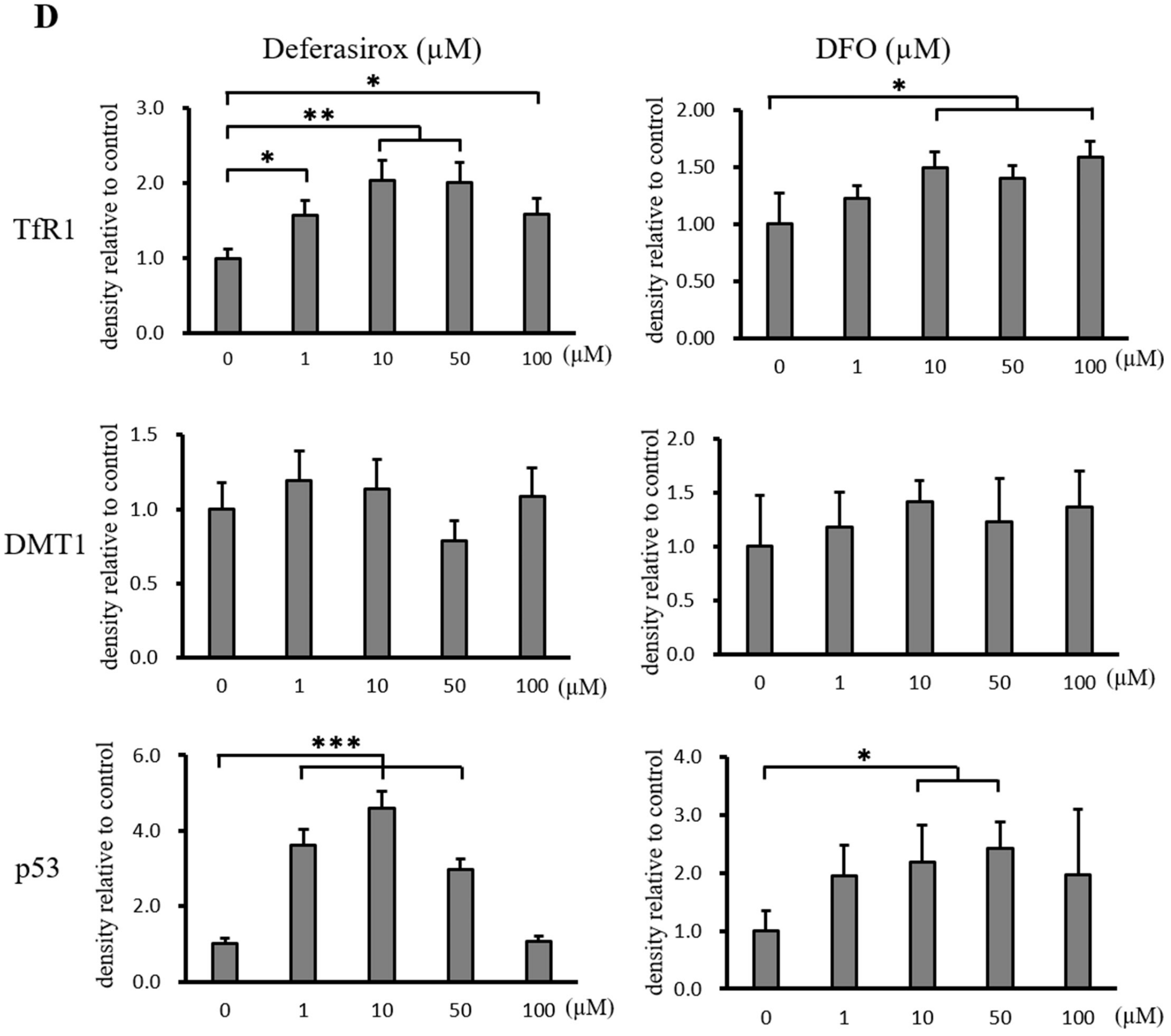

Supplementary Figure 3: (**A**) TUNEL staining of miPS-LLCcm cells. Cultured miPS-LLCcm cells were treated with 50 μM of deferasirox and DFO for 48 hours. Cells were stained using a TUNEL staining kit and DAB detection. Cells and nuclei were fragmented in the iron chelator treatment group. (**B**) Densitometry analysis of western blots showed DFO significantly decreased cyclin A2 and E1 expression. The level of cyclin A2 and cyclin E1 expressed by non-treated cells was set at 100%. Data are represented as average ± S.E.M. ($n = 3$). $^{*}p < 0.05$, $^{**}p < 0.01$, $^{***}p < 0.001$. (**C**) Western blot analysis of miPS-LLCcm cells. Cultured miPS-LLCcm cells were treated with different concentrations of deferasirox and DFO for 48 hours. Cells were then harvested and total protein was analyzed for the expression of indicated proteins. (**D**) Densitometry analysis of western blots showed the expression of TfR1 and p53 was increased by deferasirox and DFO. The expression of DMT1 was not significantly changed. Data are represented as average ± S.E.M. ($n = 3$). $^{*}p < 0.05$, $^{**}p < 0.01$, $^{***}p < 0.001$.

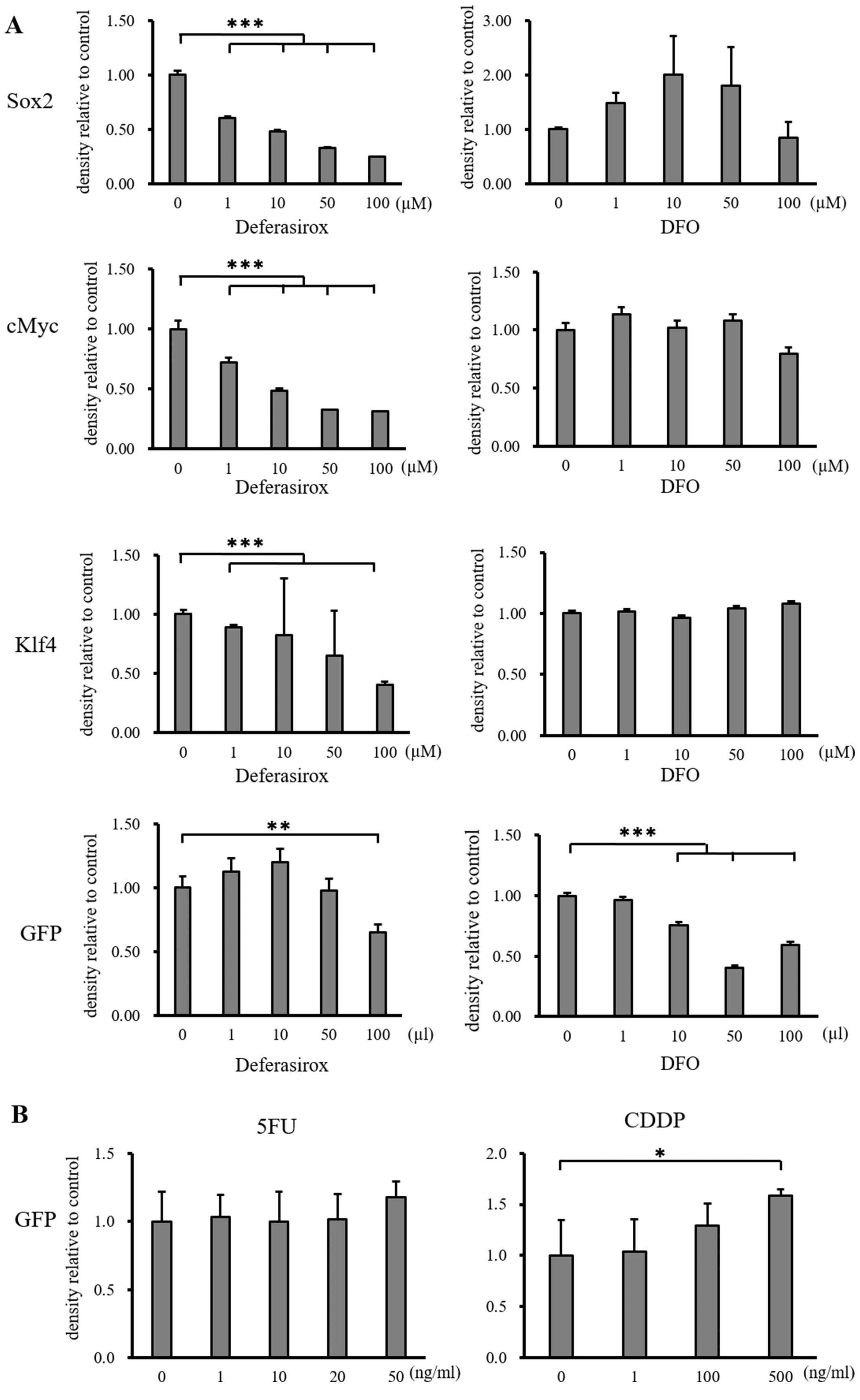

Supplementary Figure 4: (**A**) Densitometry analysis of western blotting showed Deferasirox significantly decreased Sox2, c-Myc and Klf4 expression. Deferasirox and DFO also significantly suppressed the expression of GFP. The levels of Sox2, c-Myc, Klf4 and GFP expressed by non-treated cells was set at 100%. Data are represented as average ± S.E.M. (n = 3). $^{**}p < 0.01$, $^{***}p < 0.001$. (**B**) Densitometry analysis of western blotting showed 5-FU and CDDP did not decrease the expression of GFP. The level of GFP expressed by non-treated cells was set at 100%. Data are represented as average ± S.E.M. (n = 3). $^{**}p < 0.01$, $^{***}p < 0.001$.

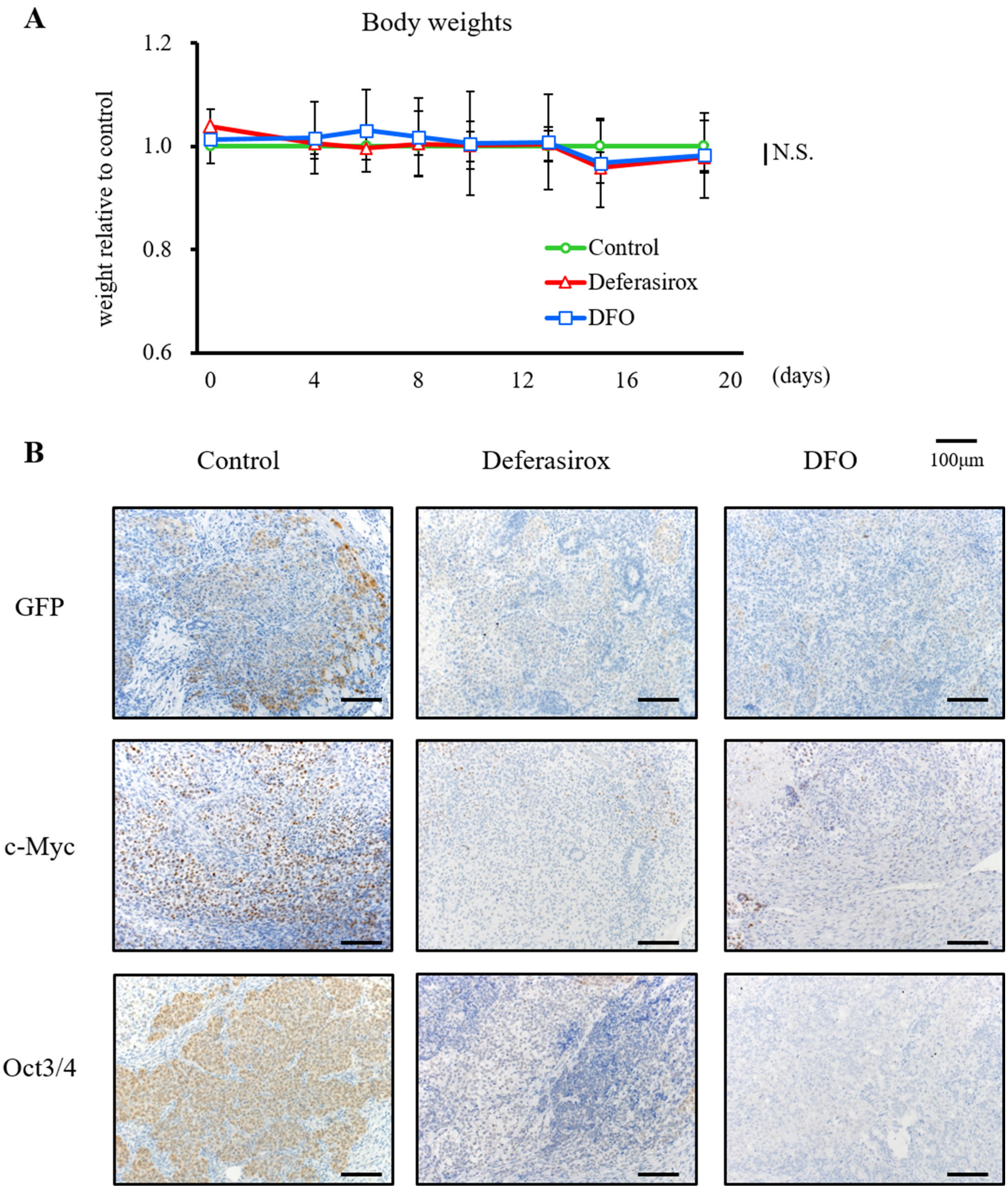

Supplementary Figure 5: (**A**) miPS-LLCcm cells (1×10^6 per animal) were implanted subcutaneously into the right back flank of mice and treatment commenced 5 days after tumor injection. Deferasirox and DFO (30 mg/kg) were locally administered by injection 5 days per week for 14 days and did not significantly affect body weight. (**B**) Resected tumors were analyzed for GFP, c-Myc and Oct3/4 expression by immunohistochemistry. The expression of GFP, c-Myc and Oct3/4 was suppressed in the deferasirox and DFO groups.
